# Prevalence and age-of-onset distributions of DSM IV mental disorders and their severity among school going Omani adolescents and youths: WMH-CIDI findings

**DOI:** 10.1186/1753-2000-3-29

**Published:** 2009-09-26

**Authors:** Sanjay Jaju, Samir Al-Adawi, Hilal Al-Kharusi, Magdi Morsi, Asya Al-Riyami

**Affiliations:** 1 Directorate of Research & Studies, Directorate General of Planning, Ministry of Health (HQ), Muscat, Sultanate of Oman; 2 Department of Behavioral Medicine, College of Medicine & Health Sciences, Sultan Qaboos University, Muscat, Sultanate of Oman

## Abstract

**Background:**

There is a dearth of studies exploring the magnitude of mental disorders amongst adolescents and youths in the Arab world. To our knowledge, this phase 2 survey in Oman is the first nationally representative school-based study to determine the prevalence of DSM-IV mental disorders (lifetime and over the preceding 12 months), their age-of-onset distributions and determine their severity over the past 12 months using the World Mental Health-Composite International Diagnostic Interview, the WMH-CIDI, used for international comparison.

**Methods:**

A total of 1,682 (91.61%) students out of 1836 students who formed the phase 2 random sub-sample of a multi-stage, stratified, random sampling design (phase 1), participated in the face-to-face structured interview using the Arabic-version of WMH-CIDI 3.0.

**Results:**

The phase 1 results using the General Health Questionnaire (GHQ-12) and Child Depression Inventory (CDI) showed depressive symptoms to be 17% prevalent in the larger sample of 5409 adolescents and youths. Amongst the phase 2 respondents from this sample, 13.9% had at least one DSM IV diagnostic label. The lifetime prevalence of Major Depressive Disorder (MDD) was 3.0%; Bipolar Mood Disorder (BMD) was 1%, Specific phobia 5.8% and Social phobia 1.6%. The female gender was a strong predictor of a lifetime risk of MDD (OR 3.3, 95% CI 1.7-6.3, *p *= 0.000); Any Mood Disorders (OR 2.5, 95% CI 1.4-4.3, p = 0.002) and Specific Phobia (OR 1.5, 95% CI 1.0-2.4, p = 0.047). The severity of illness for cases diagnosed with 12 month DSM IV disorders was found to be 80% lower in females (OR 0.2, 95%CI 0.0-0.8). The estimates over the previous 12 month period when compared with the lifetime prevalence showed a 25% to 40% lower prevalence for MDD, Specific phobia, Social phobia, Any Anxiety Disorders (AAD) and Any Mood disorders (AMD) while the rate was 80% lower for Separation Anxiety Disorder/Adult Separation Anxiety (SAD/ASA). Mood disorders were significantly lower in the 14-16 age groups (70% lower) in comparison to the older age groups and AMD showed a linear increase in prevalence across increasing age groups (*p *= 0.035).

**Conclusion:**

The implications of the present findings are not clear cut, however this study endorses the adult CIDI studies findings that mental disorders do begin earlier in life. The relatively lower prevalence of DSM IV depressive disorders cautions against any conclusive interpretation of the inflated results based on the exclusive study of the depressive symptoms alone in the same sample in the same time period. The female gender proved to be a strong predictor of lifetime risk of MDD, any mood disorder and specific phobia. Under-reporting by males or some other gender-specific factors may have contributed to such a discrepancy. The odds of the severity of illness for cases with 12 month DSM IV disorders were significantly lower in females.

## Background

Mental disorders, though difficult to conceptualize and measure, tend to contribute significantly to disability and mortality as well as an exacerbation of other medical conditions and vice versa [1]. The progress in descriptive epidemiology of Child and Adolescent Mental Disorders (CAMDs) has been hampered by a more severe version of the same measurement difficulties that plague adult studies [2]. The version 3.0 of the World Mental Health - Composite International Diagnostic Interview (WMH-CIDI) of the World Health Organization (WHO) [3] has been documented to yield reliable and valid diagnoses of mental disorders based on Diagnostic and Statistical Manual of Mental Disorders, 4th edition (DSM-IV) [4] and International Classification of Diseases, (ICD-10) [5] criteria. The use of CIDI in epidemiological and cross-cultural surveys [2] has revealed that mental disorders are among the most prevalent classes of chronic diseases in the general adult population, with lifetime-to-date prevalence often close to 50% of the population and with 12-month prevalence typically in the 12% to 25% range. In contrast to the adult findings, the estimates of the prevalence, severity and unmet need for treatment of CAMDs are imprecise. This suggests the lack of importance given to the study of CAMDs [6]. Current estimates indicate that as many as 7% to 22% of all children and adolescents are affected [7] and up to 50% of all adult mental disorders have their onset in adolescence [8]. Results from adult CIDI surveys consistently show that anxiety disorders have a median age of onset in the early to late teens, while mood disorders have a median age of onset in the early to mid twenties [2].

Oman has a population of approximately 3.5 million, with 42.7% of its inhabitants being under the age of 15. The median age of the total population is 18.8 years, it being 21.1 years for males and 16.7 years for females [9]. In 2005, more than one quarter (27.82%) of the Omani population was between 15 and 24 years of age [10]. In a nationally representative study in 2005, 17% of Omani secondary school students were found to suffer from sub-clinical depressive symptoms [11]. These symptoms are often assumed to translate into clinical depressive illness and its co-morbidities. These findings needed to be corroborated in the light of the fact that this secondary school age is a critical developmental stage in the life of an individual and carries various ramifications for the health and well-being of the individual, social cohesion, and the economic prosperity of a nation. The present study using the WMH-CIDI, aimed to determine the prevalence of DSM-IV mental disorders (lifetime and 12 month), their age-of-onset distributions and their severity (in the past 12 months cases) among the Omani adolescents and youths studying in the secondary schools.

### Methodology

#### Samples and diagnostic assessment

This study was the second phase of the two-stage epidemiological survey conducted in 2005. The respondents in both study phases were between 14 and 23 years of age [11]. The survey adopted a nationally representative, multi-stage, stratified random sampling design to select its subjects. The latest data obtained from the Ministry of Education, Oman was used as the sampling frame [12]. The sample was weighted (Table 1) based on the number of Omani secondary school students from all the different regions of Oman, their gender distribution, their classes ranging from grade 1 to grade 3 and their stream of study [13].

**Table 1:** Sociodemographic distribution of the study sample compared to population of secondary school Omani students

	**P1 Unweighted**		**P2 Unweighted**		**P1 Weighted**		**P2 Weighted**		No. of students*	
	**N**	**%**	**N**	**%**	**N**	**%**	**N**	**%**	**N**	**%**
	
Male	832	49.5	238	47.0	794.6	47.2	239.5	47.2	52167	47.2
Female	850	50.5	269	53.1	887.4	52.8	267.5	52.8	58253	52.8
**Grade**										
Grade 1	675	40.2	174	34.34	679.5	40.4	204.8	40.4	44610	40.4
Grade 2 - Art	270	16.1	79	15.58	234.3	13.9	70.6	13.9	15382	13.9
Grade 2 - Science	293	17.4	97	19.14	297.7	17.7	96.8	19.1	19544	17.7
Grade 3 - Art	216	12.8	68	13.42	214.7	12.8	57.7	11.4	13061	11.8
Grade 3 - Science	228	13.6	89	17.55	255.7	15.2	77.1	15.2	17823	16.1
**Region**										
Muscat	277	16.5	75	14.8	324.6	19.3	97.8	19.3	21306	19.3
Dhofar	197	11.7	71	14.0	153.1	9.1	46.1	9.1	10049	9.1
Al-Dakhliyah	258	15.3	82	16.2	238.6	14.2	71.9	14.2	15666	14.2
N Sharqiyah	134	8.0	52	10.3	117.1	7.0	35.3	7.0	7688	7.0
S Sharqiyah	140	8.3	41	8.1	123.9	7.4	37.3	7.4	8132	7.4
N Batinah	366	21.8	110	21.7	367.4	21.8	110.8	21.8	24121	21.8
S Batinah	193	11.5	42	8.3	223.5	13.3	67.4	13.3	14676	13.3
Al-Dhahirah	117	7.0	34	6.7	133.8	8.0	40.3	8.0	8782	8.0
**Total**	1682	100.0	507	100.0	1682	100.0	507	100.0	110420	100.0

* Source Ministry of Education data 2002

In the first phase (phase 1), a screening of 2,739 male and 2,670 female adolescents and youths (total n = 5,409) with the General Health Questionnaire (GHQ-12) and the 27 item Child Depression Inventory (CDI) was conducted.

The second phase (phase 2), which is reported here, studied a random sub-sample of 1,836 respondents from the above sample of which 1,682 (91.61%) agreed to participate in the face-to-face structured interview using the Arabic version of WMH-CIDI, PAPI (Paper and Pencil Instrument) version 3.0 [14]. This version was not validated but was professionally translated into Arabic based on a five-step process of forward translation, back translation, resolution of discrepancies between translation and back translation, pilot testing, and final revision[14]. Training for the school health doctors (interviewers) was conducted by certified CIDI qualified trainers as described elsewhere [15]. The Ministry of Education sent circulars to the sampled secondary schools to ensure their participation. The purpose of the study, its methodology and the consent forms supplied by the researchers were appended with the circular. The respective schools obtained written consent from the parents of the sampled respondents. It was clarified that the participation of their child, even after selection, was not compulsory and the parents had the choice to refuse enrollment. They were assured of confidentiality of the findings if their child was selected to be interviewed. Prior to the actual interview, a verbal consent was obtained from the respondent. The Ethics Committee of the Omani Ministry of Health approved the study.

The CIDI was administered in two parts. Part 1 included a DSM IV core diagnostic assessment screen (Part 1 sample (P1): n = 1,682; males = 832, females = 850). Part 2 included questions about risk factors, consequences, and other correlates (health service utilization, adequacy of treatment) along with assessments of additional disorders that were administered to all Part 1 respondents who met lifetime criteria for any disorder. This formed the Part 2 (P2) sample consisting of 507 respondents; males = 238, females = 269.

### Terminology

#### a) Hierarchal diagnosis

A DSM IV diagnosis hierarchy rule has been applied in this study to assess the lifetime prevalence [16] and 12 month prevalence [17] for Generalized Anxiety Disorder (GAD), Major Depressive Disorder (MDD), Dysthymia and Intermittent Explosive Disorder (IED) but not for other diagnostic labels.

#### b) Any disorder: Collapsing the rows and columns [18]

Any Mood disorder (AMD) and Any Anxiety disorder (AAD) were calculated using part 1 weights in this study (refer tables 2 and 3) while Any Impulse Control disorder (AICD) was calculated using part 2 weights. These are aggregated categories of the disorder created by collapsing the rows and/or columns in the tables for that particular category when there are too few respondents with individual disorders in that particular category. AAD includes Panic disorder, GAD, Social phobia, Agoraphobia without panic, PTSD, and SAD (Separation Anxiety Disorder)/ASA (Adult Separation Anxiety).

**Table 2:** Prevalence of DSM IV disorders

	**Total prevalence**				**Age groups**				
			
	**12 month**		**Lifetime**		**14-16**	**17-18**	**19-23**	**ChiSq^+^**	**P^&^**
			
	**N**	**%** **(SE)**	**N**	**%** **(SE)**	**%** **(SE)**	**%** **(SE)**	**%** **(SE)**		
			
***Anxiety Disorders***									
Panic disorder*	2	0.1 (0.1)	2	0.1 (0.1)	0.0 (0.0)	0.2 (0.1)	0.0 (0.0)	2.0	0.368
GAD with hierarchy*	3	0.2 (0.1)	5	0.3 (0.1)	0.4 (0.3)	0.3 (0.2)	0.0 (0.0)	4.8	0.090
Social phobia*	81	4.5 (0.5)	27	1.6 (0.3)	2.1 (0.6)	1.5 (0.4)	1.3 (0.7)	0.8	0.678
Specific phobia*	22	1.3 (0.3)	102	5.8 (0.6)	4.4 (0.9)	7.0 (0.9)	4.5 (1.2)	5.0	0.081
Agoraphobia without panic*	18	1.0 (0.2)	25	1.5 (0.3)	**1.4** **(0.6)**	**2.0** **(0.5)**	**0.3** **(0.3)**	8.8	**0.013**
PTSD **	5	0.4 (0.2)	7	0.5 (0.2)	0.0 (0.0)	0.8 (0.4)	0.5 (0.4)	4.7	0.096
SAD/ASA **	3	0.6 (0.5)	16	3.0 (1.0)	**0.2** **(0.2)**	**5.3** **(1.8)**	**0.3** **(0.3)**	7.3	**0.027**
Any Anxiety disorder^@^	54	5.6 (1.1)	79	9.0 (1.4)	5.9 (1.8)	11.3 (2.2)	7.4 (2.6)	3.5	0.171
***Mood disorders***									
MDD with hierarchy*	37	2.2 (0.4)	49	3.0 (0.4)	2.1 (0.6)	2.9 (0.6)	5.0 (1.5)	3.6	0.170
Dysthymia with hierarchy*	5	0.4 (0.2)	5	0.4 (0.2)	0.2 (0.2)	0.5 (0.3)	0.4 (0.4)	0.9	0.643
Bipolar-broad*	nc^	nc	17	1.0 (0.3)	0.5 (0.3)	1.3 (0.4)	1.0 (0.6)	2.5	0.289
Bipolar I/II/Sub threshold*	13	0.9 (0.3)	nc	nc	nc	nc	nc	nc	nc
Any Mood disorder*	53	3.3 (0.5)	69	4.3 (0.5)	**2.6** **(0.7)**	**4.6** **(0.8)**	**6.4** **(1.6)**	**6.7**	**0.035**
***Impulse control disorders***									
Conduct disorder**	2	0.2 (0.1)	2	0.2 (0.1)	0.1 (0.1)	0.1 (0.1)	0.8 (0.8)	1.8	0.406
ADHD**	3	0.1 (0.1)	4	0.2 (0.1)	0.0 (0.0)	0.2 (0.1)	0.3 (0.3)	3.7	0.162
IED with hierarchy*	24	1.5 (0.3)	31	1.9 (0.3)	2.5 (0.7)	1.5 (0.4)	2.0 (0.9)	1.5	0.472
Any Impulse control disorder**	21	3.1 (1.0)	25	3.5 (1.0)	5.9 (2.8)	2.0 (0.8)	4.4 (2.1)	2.7	0.263

*Part 1 sample, prevalence calculated using part 1 weights.

**Part 2 sample, prevalence calculated using part 2 weights.

@12 month prevalence (Part 1 weights); lifetime prevalence (Part 2 weights)

& P value is for lifetime prevalence

+ Degree of freedom is 2

^ nc = not calculated

**Table 3:** Prevalence of severity^# ^of 12 month DSM IV disorders

	Serious**		Moderate**		Mild**	
	
	**%**	**se**	**%**	**se**	**%**	**se**
	
***Anxiety disorders***						
Panic disorder*	0.0	0.0	0.0	0.0	100.0	0.0
Generalized Anxiety Disorder*	82.5	17.7	17.5	17.7	0.0	0.0
Specific phobia*	23.4	7.4	34.6	8.8	41.9	10.6
Social phobia*	43.5	15.8	56.5	5.8	0.0	0.0
Agoraphobia without panic*	76.1	18.1	16.0	13.4	7.9	7.4
PTSD**	31.3	24.2	21.6	18.0	47.1	29.1
SAD/ASA**	22.2	21.7	77.8	21.7	0.0	0.0
Any Anxiety disorder*	30.2	10.8	8.8	9.7	31.0	9.1
***Mood disorders***						
Major Depressive disorder*	45.6	14.9	43.7	12.8	10.7	5.4
Dysthymia*	27.6	23.1	72.4	23.1	0.0	0.0
Bipolar I/II/Sub threshold*	57.3	14.8	24.1	11.9	18.6	12.7
Any Mood disorder*	47.5	11.2	41.0	9.7	11.5	4.8
***Impulse Control disorders***						
Conduct disorder**	0.0	0.0	0.0	0.0	100.0	0.0
Attention Deficit Hyperactivity disorder**	52.2	31.2	15.5	16.2	32.3	28.1
Intermittent Explosive disorder*	9.9	6.5	47.8	17.4	42.3	16.7
Any Impulse control disorder**	11.0	6.3	44.0	16.3	45.0	15.6
Any Disorder**	**22.4**	**6.8**	**44.9**	**7.5**	**32.7**	**6.9**
Total sample**	**2.3**	**0.8**	**4.7**	**1.0**	**3.4**	**0.8**

# Severity for 12 month period is calculated using part 2 weights.

* Part 1 sample.

** Part 2 sample.

#### c) Severity of illness [17]

Only those respondents who met criteria for 12 month WMH-CIDI DSM IV disorder (table 2) were assessed for the 12-month severity of their disorder (Table 3). The methodological details in the WMH-CIDI related to the severity and impairment issue are available elsewhere [19]. For this analysis the following criteria defined by the Department of Health Care Policy, Harvard Medical School, Boston, USA were used and these are based on similar criteria used in a template paper [17]. It has been recommended that these be used in articles for publications.

*Severe*: either the respondent is diagnosed with a 12-month Bipolar I disorder; or the respondent has attempted suicide in last 12-months and has any 12-month diagnosis; or if the respondent has more than one 12-month diagnosis and a high level of impairment on the Sheehan Disability Scale.

*Moderate*: At least one 12-month disorder and a moderate level of impairment.

*Mild*: any 12-month disorder.

The high level of impairment was defined on a 0-10 visual analogue scale as a score of =/>8 on two out of the maximum four Sheehan Disability Scale [20] domains (home, school/work, people or social); moderate level as any domain with score =/> 4 and mild as any domain between 0 and 3.

#### d) Age of onset of mental disorders (AOO) [21]

AOO of the mental disorders was based on retrospective reporting. It may be recalled incorrectly. The AOO focuses on the onset of the syndrome, ignoring any prodrome at an earlier age.

### Statistical analysis

The data was entered in BLAISE software version 6.3.4.6, cleaned and sent to the Department of Health Care Policy, Harvard Medical School, Boston, USA for the analysis. The analysis was done by the Statistical Analysis Software (SAS) program and was based on the same statistical methodology used in the previously reported CIDI studies [[16-18,21], and [22]]. The sample distribution was compared with the national data [12] on gender, region, school grades and stream of study (science or arts) variables. The phase 1 stratification (by gender, region and grade) was done by proportional allocation and the number of classes needed was calculated for each gender, region and grade which resulted in an equal probability sample. Hence there was no need to make an adjustment for a "phase 1" weight (the school selection weight). The data were weighted to adjust for differential non-response and inclusion in the Part 2 sample. The post-stratification weights were calculated for region, by sex and by grade (details available on request). The weights were applied to all subjects in this phase two study being reported (n = 1682). There was no adjustment made for within school probability of selection, since a third of subjects within each class were selected. Prevalence and standard error of lifetime and 12 month DSM IV disorders and that within the age cohort (age at interview) are reported here as percentages. Lifetime prevalence was estimated as the proportion of respondents who were already having a given disorder up to their age at interview. Age of onset and projected lifetime risk as of age 75 years were estimated using the two-part actuarial method implemented in SAS version 8.2. The distributions of cumulative lifetime risk estimates for the disorders were standardized and examined for fixed percentiles based on the age of onset distributions (Table 4). The three age cohorts used in analysis were 14-16 years, 17-18 years and 19-23 years. Sociodemographic predictors (gender and age cohort) were examined using discrete-time survival analysis with person-years as the unit of analysis. Standard errors of prevalence estimates and survival coefficients were estimated using the Taylor series linearization method implemented in the SUDAAN software system. Multivariate significance tests were made with Wald Chi-squared tests using Taylor series design-based coefficient variance-covariance matrices. Standard errors of lifetime risk estimates were estimated using the jack-knife repeated replication method implemented in a SAS macro [17].

**Table 4:** Age at selected percentiles on the standardized age of onset distributions of disorders

**Diagnosis group**	**Disorder**	AOO* Percentile 25	**AOO Percentile 50**	**AOO Percentile 75**
Anxiety	Specific phobia	7	13	22
	Any Anxiety	8	18	22
Mood	MDD with hierarchy	16	18	19
	Any mood disorder	16	19	20
Impulse control	IED with hierarchy	13	18	20
All diagnosis	Any diagnosis	11	18	22

*Age of Onset.

The 12 month prevalence and severity were estimated by calculating means for dichotomous variables. Standard errors were obtained as above to adjust for the effects of weighting on the precision of estimates. Sociodemographic correlates were examined by transforming the predicted probabilities of class membership from the latent class analysis method into logits, the natural logarithm of the odds p_ic_/(1-p_ic_), where p_ic _is the probability that respondent i is in class c, that were then used as dependent variables in linear regression equations for effects of sociodemographic variables on the odds of class membership. Regression coefficients were exponentiated and interpreted as odds ratios with design-based 95% confidence intervals [17]. Multivariate significance was evaluated as mentioned above. The severity was calculated using Part 2 weights and as a dichotomous variable: 1 = severe or moderate, 0 = mild. All significance tests were evaluated at 0.05 with two sided tests.

## Results

### Overview

The unweighted nationally representative P1 sample (Table 1) consisted of 850 (50.5%) females and 832 (49.5%) males while the P2 sample had 269 females (53.1%) and 238 males (47.0%) participants between 14 and 23 years respectively. The sample and population distributions revealed minor differences which were corrected by post-stratification weighting. The table 1 looks at the distribution of post-stratification variables both unweighted, weighted and in the sampling frame of secondary school Omani students. The sex distribution of the part 1 (P1) sample is within 2% of the national distribution. The same is noted for the Grade 2 Art and Grade 3 Science. Region-wise this discrepancy is a maximum of up to 3%. The sex distribution for the part 2 (P2) sample, the distribution for the Grade2 Science and North Batinah region is nearly similar to that of the frame while discrepancy ranging from 1 to 6% is noted in other categories. Overall the random sub-sample fairly matches the national distribution and can be considered as a representative sample. The 14-16 years age group formed 30.38% (n = 511), 17-18 years age group 52.91% (n = 890) and 19-23 was 16.70% (n = 281). Amongst the Omani adolescents and youths surveyed (Table 5), 13.9% had one DSM IV diagnostic label, 4.5% had two or more diagnostic labels while 1.6% qualified for three or more diagnostic categories.

**Table 5:** Lifetime prevalence of having any diagnostic label

				**Age group**								
	
**Diagnostic label/DSM IV Disorder**	**Total**			**14-16 years**		**17-18 years**		**19-23 years**				
	
	**N**	**%**	**SE**	**%**	**SE**	**%**	**SE**	**%**	**SE**	**ChiSq**	**df**	**P**
Any Diagnostic label**	126	13.9	1.7	13.4	3.3	14.5	2.4	12.6	3.2	0.2	2	0.892
2+ Diagnosis**	52	4.5	0.9	3.1	0.9	5.0	1.5	5.0	2.2	1.4	2	0.501
3+ Diagnosis**	16	1.6	0.8	0.5	0.3	2.4	1.4	0.9	0.5	2.2	2	0.339

** Part 2 sample.

### Lifetime prevalence estimates (Table 2)

Broadly, the prevalence of AMD was 4.3%, AAD 9.0% and AICD was 3.5%. The prevalence of MDD was 3.0% while bipolar mood disorder (BMD) was only 1%. Only 0.4% suffered from Dysthymia. In the anxiety disorders group, the prevalence of specific phobia was 5.8%, social phobia 1.6% and post traumatic stress disorder (PTSD) 0.5%. Panic disorder and GAD were the least prevalent. SAD (ASA) had an overall prevalence of 3% while Agoraphobia without panic was 1.5%. In the impulse control disorder category, IED showed 2% prevalence. The disorders having childhood onset i.e. Attention Deficit Hyperactivity Disorder (ADHD) and Conduct Disorder were only 0.2% each.

Any mood disorder was 70% less in the younger (14-16) age group, showed linear increase in prevalence across increasing age groups and was statistically significant (*p *= 0.035). SAD (ASA) in the 17-18 age group (5.3%) was significant (*p *= 0.027) in comparison to the lower and higher age groups. Agoraphobia without panic was least in the 19-23 age group (0.3%) and was statistically significant (*p *= 0.01) across age group distribution.

### 12-month estimates (Table 2)

The estimates over the previous 12 month period when compared with the lifetime prevalence, showed a lower prevalence of 25% to 40% for MDD, specific phobia, social phobia, AAD and AMD while there was an 80% lower prevalence for ASA. The odds of being diagnosed as having a DSM IV psychiatric disorder over the previous 12 months using part 2 weights after stratification by sex and age group were not found to be significant. But when 12-month DSM IV disorder groups were considered (Table 6), the mood disorders were 70% lower in the 14-16 age groups (OR 0.3, 95% CI 0.1-1.0, chi square 7.0) in comparison to the other age groups.

**Table 6:** Correlates of 12 month DSM IV disorder groups

	**Mood**		**Anxiety**		**Impulse control**	
	
	**OR**	**95%CI**	**OR**	**95%CI**	**OR**	**95%CI**
	
***Gender***						
Male	1.0	...	1.0	...	1.0	...
Female	1.4	0.5-3.8	2.0	0.7-5.9	2.3	0.4-12.6
Chi square	0.4		1.6		1.0	
***Age***						
14-16	0.3	0.1-1.0	1.1	0.4-3.3	1.0	0.2-5.2
17-18	1.1	0.4-3.0	2.2	0.7-6.7	0.3	0.1-1.3
19-23	1.0		1.0		1.0	
Chi square	**7.0**		3.6		3.7	

### Severity of illness for 12 month disorders (Table 3)

The category wise prevalence of severity of illness in the total sample, without considering the standard errors was 2.3% serious, 4.7% moderate and 3.4% mild. In the anxiety disorder group approximately 82% of GAD and 76% of Agoraphobia without panic were classified as serious, while 78% of ASA fulfilled the moderate criteria. Around 58% of those with specific phobia and 100% of those with social phobia had severity which was between moderate and serious. All the respondents diagnosed with panic disorder were categorized as mild. Amongst the depression group, about 45% of those with MDD were equally divided between serious and moderate severity while 57% of the Bipolar I/II/Sub thresholds were classified as serious, while the majority of those with Dysthymia were classified as moderate (72%). All those with conduct disorder were categorized as mild but 45% of IED were classified as moderate and mild respectively and 55% of those with ADHD fulfilled the serious criteria. The severity of illness was 80% less in females as compared to males (OR 0.2, 95% CI 0.0-0.8, Chi square 4.9).

### Age of onset for lifetime estimates (Table 4)

The specific phobias had an earlier median age of onset at 13 years while the onset age was 18-19 years for other disorders. The mood disorders group showed a narrow age range of onset risk, the interquartile range (IQR) being 16 to 19/20 years of age. The IQR for the anxiety group was between 7-8 years and 22 years suggestive of an early age of onset distribution. The median age of onset for all the diagnoses considered together was 18 years (IQR 11-22).

### Gender as a predictor of lifetime risk (Table 7)

**Table 7:** Gender as the predictor of lifetime risk

	**Female**			
	**OR**	**95% CI**	**Chi Sq** **(df = 1)**	**P**

Specific phobia	**1.5**	1.0-2.4	3.9	**0.047**
Anxiety disorder	1.2	0.6-2.4	0.3	0.602
Major Depressive Disorder	**3.3**	1.7-6.3	12.8	**0.000**
Any Mood disorder	**2.5**	1.4-4.3	9.5	**0.002**
Intermittent Explosive disorder	2.5	1.0-6.1	3.7	0.055
Any disorder	1.6	0.9-2.9	3.0	0.085

Female gender has been found to be a strong predictor of lifetime risk of MDD (OR 3.3, 95% CI 1.7-6.3, *p *= 0.000); AMD (OR 2.5, 95% CI 1.4-4.3, p = 0.002) and specific phobia (OR 1.5, 95% CI 1.0-2.4, *p *= 0.047).

## Discussion

The World Mental Health Survey Initiative 2000 conducted CIDI surveys between 2001 and 2005 in different countries of which 17 study findings have been published [22]. The study cohorts belonged to the age groups of 18 years and above. Lebanon and Israel were the only two countries which reported from the Middle East. The present study in the same time period, to our knowledge, was the first CIDI survey among the adolescents and youths between 14 and 23 years in this region. This cohort closely resembles the New Zealand study conducted during a similar time period which included those aged 16+ years though only the 18+ age group was analyzed.

The use of WHO-CIDI is justified as it is the only available instrument based on extensive cross-national field trials. The adult CIDI surveys have consistently shown that anxiety disorders have a median age of onset in the early to late teens, while mood disorders have a median age of onset in the early to mid twenties [2]. The 18+ age group respondents in this study contributed only 16.70% to the overall sample but still the results of adult studies have been used for discussion in the present paper, in spite of inherent limitations, so as to help corroborate the findings in the preceding statement and also facilitate international comparisons based on one standardized instrument. Hence the findings of other studies which have used different instruments on the respondent sample similar to ours have been avoided in discussion as far as possible.

The response rate in the present study was 91.61%. In the literature, it ranged between a minimum 45.9% in France and a maximum 87.7% in Columbia while the respective response rates were 70% for Lebanon, Israel 72.6%, and New Zealand 73.3% [22]. The higher compliance in the present study may be due to the fact that the study was conducted with the constraints and advantages of a school setting. It is possible that the response rate from community-based surveys rather than school-based ones would strongly hinge on the adults' choice to either participate in the study or not. Therefore, it appears that a community survey tends to have a lower response rate.

The initial finding of a prevalence of 17% of depressive symptoms based on self reported CDI [11] in the source sample translated during the same time period to markedly lower estimates of lifetime prevalence of MDD and BMD. Studies in adolescents and youth using different research methodologies have reported variable estimates of depressive symptoms from about 9% to between 25% and 40% [23,24]. The present study suggests that one need not be alarmed by results based on depressive symptoms only but it endorses the adult studies findings that mental disorders do begin earlier in life. A vast majority of adults with serious mental disorders experience a combination of panic, generalized anxiety, depression, phobia and substance abuse which differ substantially in their ages of onset. Anxiety, oppositional-defiant and attention-deficit problems typically have earlier ages of onset. It is hypothesized that the cumulative effect of these disorders could be of causal significance and hence measures need be taken to reduce the prevalence of serious mental disorders in adolescents and youths [25].

In the prevalence estimates over previous 12 month period, lower prevalence is noted for MDD, specific phobia, ASA and any anxiety and any mood category as compared to the lifetime prevalence suggestive of an earlier onset. The decrease could perhaps be attributed to natural causes or available treatment. The linear increase seen in the prevalence of any mood disorders in the lifetime estimates confirms the earlier findings that mood disorders have a later age of onset [26].

The age of onset distributions for lifetime estimates overlap with the findings of adult studies from other countries [22] necessitating the targeting of this population in Oman for further investigation and intervention if necessary. The results corroborate with other studies which show that impulse control disorders have the earliest age of onset distributions, an early median age of onset and a narrow age of onset risk between 13 and 21 years. The estimation was not done for the other subgroups, as the respondents were fewer than 30 in the study sample. The median age of onset for specific phobia was 13 years and well within the 7-14 years range reported. But the IQR of 7-22 years in this study varied from the narrow IQR of 8-11 years in other studies. This could be due to a relatively smaller sample size in a limited age range. The other anxiety subgroups had a later median age of onset (median 25-50 years, IQR 31-41) in adult surveys. This study showed a similar trend, with a later median age of onset at 18 years (IQR 8-22 years) for the any anxiety category when compared to specific phobias. The difference between the impulse control group and phobias in comparison with the other anxiety disorder groups can be attributed to wider cross-national variations in the latter. This must be interpreted with caution due to the methodological considerations [22]. The discrepancy in IQR for specific phobia in this study compared to others could be due to the same reason. For mood disorders, the reported prevalence is consistently low until the early teens, at which time a roughly linear increase begins that continues through the late middle age, with a more gradual increase thereafter and this study results are on similar lines.

Studies seem to indicate that the odds ratios for anxiety and mood disorders are higher in the recent cohorts compared to the older cohorts [22]. These studies compared each cohort of approximately 15 years and consisted of 4 cohorts ranging from 18 years to 65+. This study being restricted to three narrow cohorts of 14-16, 17-18 and 19-23 years perhaps did not exhibit the above trend.

The female gender proved to be a strong predictor of lifetime risk of MDD, AMD and specific phobia. A differential willingness hypothesis has been proposed as a plausible explanation of the observed finding that women report higher rates of anxiety and depression than males who tend to under report, thus leading to biased estimates [27]. Alternatively, there may be gender-specific factors that contribute to such a discrepancy, but it is beyond the scope of discussion in this paper.

This study demonstrated a significant increase in lifetime prevalence in agoraphobia without panic and SAD/ASA in the 17-18 year age group compared to the lower and higher age groups. Cross-nationally these disorders show an inverted U-shaped trend and tend to decrease as age increases [2]. The estimates of mild and moderate severity in the anxiety spectrum in this study appear to contradict the above findings due to this study being restricted to three narrow cohorts in a restrictive age range. Also, as the anxiety spectrum illnesses tend to be characterized by somatic distress, it is possible that cultural factors may have played a part in the present trend. Further exploration into this phenomenon is therefore warranted. The rates for lifetime prevalence in the three classes of having any disorders are comparable to other cross national studies [22] which suggest the IQR of 9.9-16.7% for any anxiety disorders and the IQR of 3.3-21.4% for any mood disorders. The younger cohort in our study could account for lower bound estimates of prevalence rates. The Impulse control disorders are comparably least prevalent (IQR 3.1-5.7%) across countries and our sample showed a similar trend for lifetime prevalence. The narrow age groups of the respondents in our study accounts for a limited age range of onset risk in the mood disorders group.

The WMH measures of severity were applied only to 12 month cases as there is at present no way to estimate the severity of lifetime cases. The prevalence of severity is quite similar to the available findings in other countries where the majority of cases between 33% and 90% (IQR 40-53%) were rated mild [28], but even mild cases could be impairing and evolve into more serious disorders over time [29]. The odds of severity were 80% less in females which can be attributed to their willingness to discuss personal problems which had a cathartic effect and hence reduced the severity of the problem. It has been noted that individuals who air their emotional experiences are likely to have positive health outcomes [30]. The no difference in the odds of severity across age groups was because of the narrow age range in this study.

A recent review of the magnitude of mental disorders in children and adolescents from recent community surveys across the world demonstrated that though there is substantial variation in the results depending upon the methodological characteristics of the studies, the findings demonstrate that approximately one fourth of youths experienced a mental disorder during the past year, and about one third across their lifetimes. Anxiety disorders are the most frequent conditions in children, followed by behavior disorders and mood disorders [31]. A similar trend was noted in this study. Belfer, reporting the findings of different researchers', states that children with depression, ADHD and conduct disorder have higher rates of health care utilization, impose costs on society in terms of education, and are a burden on the criminal justice system and on social services [8]. In 2002, only 7% of the countries worldwide (14 out of 191) had a clearly articulated specific child and adolescent mental health policy [32]. Ironically, the countries with the highest proportion of children and adolescents in their populations are those countries that are most likely to lag behind in child and adolescent mental health policy [33].

## Suggestions

The situation in Oman, in spite of findings with lower bound estimates, does present a cause for concern, considering the majority of the population is still in adolescence and youth. If the present findings can withstand further scrutiny, Oman needs to institute an informed agenda for the welfare of its adolescents and youth vis-à-vis mental health policy. A longitudinal study in Oman to assess adult outcomes of adolescent psychological problems will be essential if not paramount to set in motion evidence-based policy and services for people with mental illness. Similarly there is an urgent need to estimate the burden of mental problems amongst adults in Oman since strong evidence has emerged from adult studies that mental disorders have much earlier ages of onset than other chronic diseases. An awareness of mental problems amongst parents, teachers and students by health educators and the media could assist in addressing the stigma of mental illness and limit the tendency to under-report mental distress or not to report it at all. This could ensure better participation with proper understanding in such surveys to yield reliable estimates.

## Limitations

The present findings are discussed with some of the possible limitations that are often an integral part of such studies which have a cross-sectional design. The ecological validity would have been heightened if the information elicited were corroborated across a range of situations including opinions from parents and teachers. Similarly, it is possible that the present survey may have omitted those who had dropped out from school as a result of mental ailments and also those who were non-school going for other reasons. The lower bound estimates of mental illness among secondary school respondents in Oman merits some speculation. Mental disorders carry stigma worldwide including in Oman [34] and hence concerns have been raised about under-reporting as a serious problem in any epidemiological survey [35]. The estimated lifetime prevalence of having any disorder varied widely from 47.4% in United States to 12% in Nigeria, with lower estimates in developing countries such as Peoples' Republic of China. Other countries which reported lifetime prevalence estimates are Lebanon 25.8%, Israel 17.6% and New Zealand 39.3% [22]. Around 14% of our sample has at least one lifetime diagnostic label which is below the 25th percentile observed across other countries which had IQR between 18.1-36.1%. The cultural teachings that tend to perceive psychiatric distress as physical illness may have contributed to reporting bias. The variable estimates could also be due to interviewer errors as noted in other surveys because the interviewers rushed through the interviews as they were paid per interview rather than hourly [22]. This could hold true for our study even though the payment was made per month. However in this present study it was difficult to ascertain whether the students' unwillingness to report symptoms could have been affected due to the presence of the school health doctor who conducted the interview. Along with this factor, the questionnaire, being too detailed and time consuming, could have caused fatigue in the respondents of this age group, perhaps resulting in negative replies. It has been reported that the prevalence of emotional problems reported in epidemiological surveys should be considered lower bound estimates rather than accurate reflections of the true prevalence in the population [36]. The other possibility that these young respondents may have given some affirmative replies without really understanding the content of the questions or without knowledge of its implications just to satisfy the interviewer in the form of a person of authority cannot be denied. This and the interviewer errors mentioned above could also result in inflated estimates.

Much of the emotional turbulence especially during the adolescence period is known to resolve in adulthood [2]. Costello et al [37] recommend that caution should be exercised about the clinical significance of less serious symptoms unless longitudinal studies prove that these are associated with a risk of future clinically significant disorders. On the other hand, there is evidence to suggest that single disorders often progress to complex co-morbid disorders that are impervious to treatment and more likely to recur than less complex conditions [25]. Therefore, our subjects need to be re-assessed at a later period for a meaningful understanding of the impact of the present labeling.

## Conclusion

The younger cohort in our study could account for lower bound estimates of prevalence rates in comparison to adult findings. The implications of the present findings are not clear cut, however this study endorses the adult CIDI studies findings that mental disorders do begin earlier in life. The relatively lower prevalence of depressive disorders cautions against being alarmed by results based on studies of depressive symptoms on the same sample in the same time period.

The female gender proved to be a strong predictor of lifetime risk of MDD, AMD and specific phobia. The odds of severity of illness were significantly less in females, perhaps due to their willingness to discuss personal problems which has a cathartic effect. Under-reporting by males or some other gender-specific factors may contribute to such a discrepancy and needs to be investigated.

## Abbreviations

AAD: Any Anxiety Disorders; ADHD: Attention Deficit Hyperactivity Disorder; AICD: Any Impulse Control Disorders; AMD: Any Mood Disorders; AOO: Age of onset of mental disorders; ASA: Adult Separation Anxiety; BMD: Bipolar Mood Disorder; CAMDs: Child and Adolescent Mental Disorders; CDI: Child Depression Inventory; CIDI: World Mental Health-Composite International Diagnostic Interview; DSM IV: Diagnostic and statistical manual of mental disorders, 4^th ^edition.; GAD: Generalized Anxiety Disorder; GHQ-12: General Health Questionnaire; ICD 10: International classification of diseases; IED: Intermittent Explosive Disorder; IQR: Interquartile range; MDD: Major Depressive Disorder; P1: Part1 sample; P2 sample: Part 2 sample; PAPI version: Paper and Pencil Instrument version; PTSD: Post traumatic stress disorder; SAD: Separation Anxiety Disorder; SAS: Statistical Analysis Software; SPSS 9.0: Statistical Package for Social Sciences 9.0; WMH-CIDI: World Mental Health - Composite International Diagnostic Interview; WMH: World Mental Health Survey; WHO: World Health Organization.

## Competing interests

The authors declare that they have no competing interests.

## Authors' contributions

SJ interpretation of data analysis, drafting the manuscript and its revision of for intellectual content; SAA revision of manuscript for intellectual content; HAK, MM & AAR conception, design and acquisition of data.
